# Economic evaluation alongside the Speed of Increasing milk Feeds Trial (SIFT)

**DOI:** 10.1136/archdischild-2019-318346

**Published:** 2020-04-02

**Authors:** Warda Tahir, Mark Monahan, Jon Dorling, Oliver Hewer, Ursula Bowler, Louise Linsell, Christopher Partlett, Janet Elizabeth Berrington, Elaine Boyle, Nicolas Embleton, Samantha Johnson, Alison Leaf, Kenny McCormick, William McGuire, Ben J Stenson, Ed Juszczak, Tracy E Roberts

**Affiliations:** 1 University of Birmingham, Birmingham, UK; 2 Division of Neonatal-Perinatal Medicine, Dalhousie University—Faculty of Medicine, Halifax, Nova Scotia, Canada; 3 National Perinatal Epidemiology Unit, Nuffield Department of Population Health, University of Oxford, Oxford, Oxfordshire, UK; 4 National Perinatal epidemiology Unit, University of Oxford, Oxford, UK; 5 Faculty of Medicine & Health Sciences, University of Nottingham, Nottingham, Nottinghamshire, UK; 6 Neonatology, Royal Victoria Infirmary, Newcastle upon Tyne, UK; 7 Department of Health Sciences, University of Leicester, Leicester, UK; 8 Newcastle University, Newcastle upon Tyne, UK; 9 University of Leicester, Leicester, UK; 10 Neonatal Medicine, Southmead Hospital, Bristol, UK; 11 John Radcliffe Hospital, Oxford, Oxfordshire, UK; 12 Centre for Reviews and Dissemination, University of York, York, North Yorkshire, UK; 13 Neonatology, Neonatal Unit, Simpson Centre for Reproductive Health, Royal Infirmary of Edinburgh, Edinburgh, UK; 14 Nuffield Department of Population Health, University of Oxford, Oxford, UK; 15 Health Economics Unit, Institute of Applied Health Research, University of Birmingham, Birmingham, UK

**Keywords:** health economics, infant Feeding, neonatology

## Abstract

**Objective:**

To evaluate the cost-effectiveness of two rates of enteral feed advancement (18 vs 30 mL/kg/day) in very preterm and very low birth weight infants.

**Design:**

Within-trial economic evaluation alongside a multicentre, two-arm parallel group, randomised controlled trial (Speed of Increasing milk Feeds Trial).

**Setting:**

55 UK neonatal units from May 2013 to June 2015.

**Patients:**

Infants born <32 weeks’ gestation or <1500 g, receiving less than 30 mL/kg/day of milk at trial enrolment. Infants with a known severe congenital anomaly, no realistic chance of survival, or unlikely to be traceable for follow-up, were ineligible.

**Interventions:**

When clinicians were ready to start advancing feed volumes, infants were randomised to receive daily increments in feed volume of 30 mL/kg (intervention) or 18 mL/kg (control).

**Main outcome measure:**

Cost per additional survivor without moderate to severe neurodevelopmental disability at 24 months of age corrected for prematurity.

**Results:**

Average costs per infant were slightly higher for faster feeds compared with slower feeds (mean difference £267, 95% CI −6928 to 8117). Fewer infants achieved the principal outcome of survival without moderate to severe neurodevelopmental disability at 24 months in the faster feeds arm (802/1224 vs 848/1246). The stochastic cost-effectiveness analysis showed a likelihood of worse outcomes for faster feeds compared with slower feeds.

**Conclusions:**

The stochastic cost-effectiveness analysis shows faster feeds are broadly equivalent on cost grounds. However, in terms of outcomes at 24 months age (corrected for prematurity), faster feeds are harmful. Faster feeds should not be recommended on either cost or effectiveness grounds to achieve the primary outcome.

**What is already known on this topic?**

- Economic evidence regarding enteral feeding regimes is scarce.
- Previous randomised controlled trial (RCT) evidence was based on short-term outcome data.
- The Speed of Increasing milk Feeds Trial was the largest enteral feeding regime trial and the first to report neurodevelopmental outcomes at 2 years of age.

**What this study adds?**

- This is the first economic evaluation of enteral feeding practices alongside a RCT.
- Increasing milk feed volumes at a faster rate in very preterm or very low birth weight infants is not a cost-effective strategy.
- The costs and consequences of faster feeds in the long term are likely too high to recommend this as routine clinical practice.

## Introduction

The total cost of preterm birth to the public sector is nearly £3 billion over childhood, of which 34% is attributable to very preterm birth before 32 weeks of gestation.^1^ Costs arise from increased healthcare resource utilisation such as hospital inpatient and outpatient care, community care and medications.^2^ There are also considerable societal costs associated with preterm birth-related morbidities, such as productivity loss resulting from time taken off work by parents and carers and additional expenditure on home adjustments, special equipment and travel.^2 3^ There is uncertainty regarding enteral feeding practices of very preterm and very low birth weight (VLBW; <1500 g) infants. Increasing milk volumes slowly is associated with increased risk of late onset sepsis (LOS),^4–6^ while faster increases may increase the likelihood of necrotising enterocolitis (NEC).^7^ In very preterm or VLBW infants, LOS and NEC are important causes of long-term neurodevelopmental disability.^7 8^ Children with neurodevelopmental disabilities require a range of services beyond simply healthcare, such as social services, special educational support and rehabilitation^9^ and costs accrue throughout the individual’s lifespan. The Speed of Increasing milk Feeds Trial (SIFT) aimed to address uncertainty in enteral feeding practices by comparing two rates of enteral feed advancement (18 vs 30 mL/kg/day) in very preterm and VLBW infants.^7^ This paper presents the economic evaluation undertaken alongside SIFT.

## Methods

Details of the trial have been published elsewhere.^7^ Briefly, 2804 babies were recruited into the trial, of which 1400 were randomised to faster feeds and 1404 were randomised to slower feeds. Outcomes were assessed until discharge home from neonatal units and again at age 24 months, corrected for prematurity (see online supplementary material figure S1). Resource use data were collected prospectively from centres participating in the trial. All centres completed a total of eight different data collection forms that included specific items measuring healthcare use. Where serious adverse events were reported, the associated resource use was collected on an additional form by the relevant participating centres. Health service use for the first 2 years of life was also measured through a parent questionnaire, which included healthcare-related resource use items such as use of primary care services and hospital readmissions.^7^

10.1136/archdischild-2019-318346.supp1 Supplementary data

## Costs

A National Health Service and Personal Social Services perspective was adopted; thus, only the direct costs to the health service provider during the trial’s duration were considered.

Table 1 presents the relevant items of resource use, their associated unit costs and the source from which these costs were obtained. All costs were expressed in pounds sterling (GBP) and in 2017–2018 prices. Costs were inflated where necessary, using the hospital and community health services index.^10^ More detail on resource use and disaggregated costs is presented in the online supplementary tables S1 and S2.

10.1136/archdischild-2019-318346.supp2 Supplementary data

**Table 1:** Unit costs of resource items (2017–2018 prices)

Resource use items	Unit cost (£)*	Source
Intervention		
Cost per day on parenteral nutrition	45	Walter *et al* 28
Intensive care		
Cost per day in intensive care (differentiated by level of care required)		NHS Reference Costs^29^
Level 1—Intensive Care	1295	
Level 2—High Dependency Care	1032	
Level 3—Special Care	510	
Initial hospital stay		
Cost per pulmonary haemorrhage	1485	NHS Reference Costs^29^
Cost per IVH by severity:		
Grade 1 IVH/Germinal matrix haemorrhage	862	NHS Reference Costs^29^
Grade 2 IVH	1472	NHS Reference Costs^29^
Grade 3/4 IVH	1519	NHS Reference Costs^29^
Course of shunts for hydrocephalus	2608	NHS Reference Costs^29^
Bronchopulmonary dysplasia	5954	NHS Reference Costs^29^
Periventricular leukomalacia	1341	NHS Reference Costs^29^
Retinopathy treated medically or surgically	1603	NHS Reference Costs^29^
PDA treated with NSAID	1152	BNFC^30^
Surgeries due to gut signs	6629	NHS Reference Costs^29^
Cost of antibiotic medication per day	3.00	BNFC^30^
Cost of antifungal treatment per day	1.06	BNFC^30^
Cost per mL of Preterm milk formula	0.02	Ganapathy *et al* 31
Cost per packet of breast milk fortifier	0.93	Ganapathy *et al* 31
Cost per litre of donor breast milk	335	Renfrew *et al* 32
Cost per 200 mL of term formula milk	2.00	Renfrew *et al* 32
Resource use during 2-year follow-up		
Cost per out-patient day	199	NHS Reference Costs^29^
Cost per in-patient day	635	NHS Reference Costs^29^
Cost per operation	2247	NHS Reference Costs^29^
Cost per GP visit	33	Curtis and Burns^10^
Cost per Health Visitor visit	75	Curtis and Burns^10^
Cost per Community Nurse visit	36	Curtis and Burns^10^
Cost per Home Visitor/Volunteer visit	19	Curtis and Burns^10^
Cost per Community Paediatrician visit	407	NHS Reference Costs^29^
Cost per Physiotherapist visit	95	NHS Reference Costs^29^
Cost per Social Worker visit	39	Curtis and Burns^10^
Cost per Speech and language therapist visit	95	NHS Reference Costs^29^
Cost per Dietician visit	85	NHS Reference Costs^29^
Cost per Other health professional visit	135	NHS Reference Costs^29^

*Inflated to 2017–2018 costs using the UK hospital and community health services pay and prices index. Costs were assigned using a macrocosting (top-down) approach.^20^

GP, general practitioner; IVH, intraventricular haemorrhage (intracranial abnormality); NSAID, non steroidal anti-inflammatory drugs; PDA, patent ductus arteriosus.

## Economic analysis

A preliminary cost-consequence analysis was conducted to compare the disaggregated costs and outcomes for both feeding increments.^11^ Mean costs per infant were estimated for each arm and the mean cost differences between the two feeding allocations were calculated. A bootstrapping approach with replacement was undertaken to calculate CIs around the mean costs.^12^

The primary base case economic analysis took the form of a cost-effectiveness analysis, a method for assessing the gains in health relative to the costs of the different health interventions.^13^ Costs and clinical outcomes associated with each feeding allocation were combined by calculating incremental cost-effectiveness ratios (ICERs). Cost data were discounted at 3.5% but discounting is not applied to outcomes in natural units. Cost-effectiveness was based on the principal outcome of additional cost per survivor without disability at 24 months corrected age.

## Stochastic cost-effectiveness analysis

A stochastic cost-effectiveness analysis (PSA) was conducted on the base-case results. The approach taken in the PSA is that all important variables relating to costs and clinical outcomes are given a distribution that describes the uncertainty surrounding the mean. The distributions are simulated 5000 times. Each time, random numbers are drawn from the appropriate distributions. After each simulation, the incremental costs and effects are plotted in a cost-effectiveness plane which comprises four quadrants: north-east (NE), north-west (NW), south-east (SE) and south-west (SW). The scatterplot that is produced represents the simulations. If dots from the scatterplot are in the NE quadrant, this indicates that the intervention is more costly and more effective compared with the comparator (slower feeds). Dots in the NW quadrant indicate that the intervention is more costly and less effective than the comparator. Based on these simulations, the probability that the intervention would be cost-effective is presented. This is the standard approach for health economics following accepted guidelines (CHEERS)^14^ and is a presentation of results of cost-effectiveness studies which would be required by decision-makers such as NICE.^15^

Results were also presented using cost-effectiveness acceptability curves (CEACs) to reflect sampling variation and uncertainties in the cost-effectiveness value where appropriate. A CEAC shows the probability that an intervention (eg, faster feed increments) is cost-effective compared with the alternative (eg, slower feed increments), given the observed data, for a range of maximum monetary values (thresholds) that decision-makers might be willing to pay for a particular unit change in outcome.^16^ We examined cost-effectiveness across a range of monetary willingness to pay thresholds, 0–£40 000 per additional survivor without disability at 24 months corrected age. To account for missing resource use data where follow-up data were not available, multiple imputation was performed following published and widely implemented methodology.^17 18^ We used the multiple imputation technique of predictive mean matching to impute the missing values for total costs at 2 years. The imputation model was based on treatment group, and used M=20 imputed datasets.

## Results

### Participants

A total of 2804 infants were recruited, 1400 of which were randomised to faster feeds and 1404 to slower feeds. The proportions of infants withdrawn from the trial or lost to follow-up are presented in online supplementary figure S1. The data on the primary outcome at 24 months were available in 1224 (87.4%) of the faster group and 1246 (88.7%) of the slower group. The primary base case analysis comprised of only those infants for whom there was complete outcome data.

### Resource use and costs

The number of parents who fully completed the resource use questionnaire was 842/1224 (68.8%) and 873/1246 (70%) in the fast and slow feeding increment arms of the trial, respectively. Average volumes of resource use and costs per infant during initial hospital stay and postdischarge up to 2 years corrected age are detailed in online supplementary tables S1 and S2.

There was very little variation in resource use during initial hospital stay and postdischarge between groups. Postdischarge health service costs were generally higher for the faster increment, particularly for primary care services such as general practitioner, health visitor and community nurse visits.

The mean costs for each group are presented in table 2. The cost for faster feeds was, on average, an estimated £242 less per infant (95% CI −6307 to 6251) than slower feeds during infants’ initial hospital stay. However, during the 2 year follow-up, the faster increment group was more costly, on average, by approximately £510 (95% CI −2864 to 4508) per infant. In terms of costs up to 24 months, faster feeds were slightly more costly by, on average, £267 (95% CI 6928 to 8117) per infant.

**Table 2:** Mean total costs (£s sterling, 2016–2017 prices)

Cost category	Faster increments (n=1224)			Slower increments (n=1246)			
	Total cost	Mean	SD	Total cost	Mean	SD	Bootstrap mean difference (95% CI)
Costs of initial hospital care	124 386 552	101 623*	80 759	126 923 790	101 865	80 498	−242 (−6307 to 6251)
Costs from initial discharge from hospital up to 24 months corrected age	10 151 856	8294	49 585	9 698 864	7784	38 473	510 (−2864 to 4508)
Total costs of health service use after initial discharge from hospital and up to 24 months corrected age†	134 538 408	109 917	98 040	136 623 900	109 650	94 788	267 (−6928 to 8117)

*Mean total costs are calculated as total cost for this category divided by the sample size of the arm (eg, 124 386 552/1224 for costs of initial hospital care in the faster feeding arm).

†Total costs are the sum of costs of initial hospital care and costs from discharge up to 24 months corrected age.

### Cost-consequence analysis

In terms of overall costs, the average cost per infant for faster feeds was slightly more costly than slower feeds (mean difference £267, 95% CI −6928 to 8117).

Although there were fewer deaths in the faster feed group, for the primary outcome of survival without moderate or severe neurodevelopmental disability at 24 months corrected age, the intervention (faster feeds) was less effective than the comparator (slower feeds). Increasing milk feeds at a faster rate was associated with 46 (2.6%) more infants with moderate to severe disability compared with the slower feed group (mean difference: 0.96, 95% CI 0.92 to 1.01).

The breakdown of costs shows that faster feeds compared with slower feeds is slightly less costly, on average, during infants’ initial hospital stay, but in the postdischarge period up to 24 months, the 2-year follow-up data show that costs are greater in those allocated faster increments, on average. This resulted in a slightly higher mean total cost per infant in the intervention arm (faster feeds). This is due to greater resource use associated with faster increments, in terms of hospital inpatient stays and primary care visits in the postdischarge period (online supplementary table S1 and S2).

### Cost-effectiveness analysis

**Figure 1:**
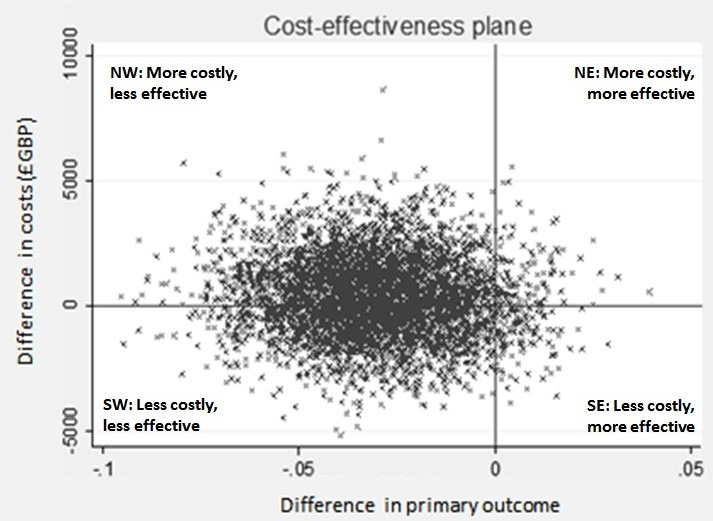
Cost-effectiveness plane (fast vs slow feeds). NE, north-east; NW, north-west; SE, south-east; SW, south-west.

Faster feeds are shown to cost more per infant on average and are also less effective in achieving the primary outcome; thus, they are dominated by the comparator (slower feeds) (see table 2, table 3, figure 1). There is therefore no ICER to present in this circumstance.

**Table 3:** Cost-consequence and cost-effectiveness analyses

Costs/Consequences	Faster increments (n=1224)	Slower increments (n=1246)
Total costs of health service use for 2 years	134 538 408	£136 623 900
Costs of initial hospital care	£124 386 552	£126 923 790
Costs from initial discharge from hospital up to 24 months corrected age	£10 151 856	£9 698 864
Survival at hospital discharge	1332/1394 (95.57%)	1337/1399 (95.23%)
Death before discharge home	60/1392 (4.3%)	65/1393 (4.7%)
Survival without moderate or severe neurodevelopmental disability at 24 months	802/1224 (65.5%)	848/1246 (68.1%)
Survival (at 24 months, corrected age)	1326/1394 (95.1%)	1322/1399 (94.5%)
Moderate to severe neurodevelopmental disability	473/1394 (33.9%)	405/1399 (28.9%)
Neonatal late onset invasive infection (LOII)	414/1389 (29.8%)	434/1397 (31.1%)
NEC	70/1394 (5.0%)	78/1399 (5.6%)

Disability was defined as moderate/severe in any of the following categories any of: moderate/severe visual impairment, moderate/severe hearing impairment, moderate/severe gross motor impairment or moderate/severe cognitive impairment.

NEC, necrotising enterocolitis.

### Stochastic cost-effectiveness analysis

**Figure 2:**
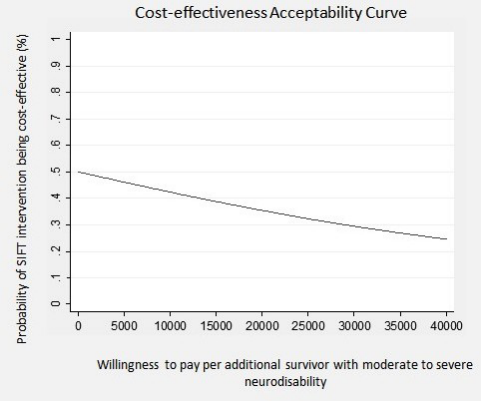
Cost-effectiveness acceptability curve (fast vs slow feeds). SIFT, Speed of Increasing milk Feeds Trial.

The cost-effectiveness plane for the base-case analysis is presented in figure 1. The majority of the scatterplots (depicting paired incremental costs and outcomes) are in the NW and SW quadrants. Scatterplots falling in the NW quadrant represent higher cost and worse outcome than the comparator, while scatterplots in the SW quadrant represent worse outcome and lower costs. Thus, figure 1 suggests that faster feed increments are a less effective intervention. However, it is uncertain whether faster feeds are likely to be more costly (NW) or less costly (SW) relative to slower feed increments. The CEAC (figure 2) shows the probability of faster feeds being cost-effective at various values of decision-makers’ willingness-to-pay per additional survivor without moderate to severe disability at 24 months of age (corrected for prematurity). The CEAC (figure 2) suggests that the probability of the intervention being cost-effective is less than 50% for all willingness to pay values. In addition, the probability of cost-effectiveness decreases as the willingness to pay increases.

Mean total costs for all infants, adjusting for missing data using multiple imputation, are found in the online supplementary table S3. When the missing values were accounted for, faster feed increments remain more costly in comparison to slower feed increments but at a slightly higher level (£378 more) per infant, reflecting the high level of uncertainty in the difference in costs, especially with regard to the healthcare resource use after discharge estimated by the multiple imputation.

## Discussion

The results of the economic evaluation suggest that for very preterm or VLBW infants, daily increments in milk volumes at a faster rate (30 mL/kg/day) are per infant more costly and less effective in comparison to a slower rate (18 mL/kg/day).

In terms of initial hospital and postdischarge costs between the two feeding increments evaluated, a faster increment (30 mL/kg/day) was shown to be slightly more costly, on average, compared with feeding with a slower increment (18 mL/kg/day) at 2 years corrected age. The average cost per infant for faster increments was £109 917 compared with £109 650 for slower increments. However, in terms of the clinical outcomes, faster feeds were less effective than slower feeds (18 mL/kg/day) in achieving the principal outcome of survival without moderate or severe neurodevelopmental disability at 24 months corrected age. Fewer infants in the faster increments arm achieved the primary clinical outcome (n=802) compared with the slower increment arm (n=848).

When the uncertainty around all the estimates is incorporated into the analysis, the results suggest that the faster feeding increment is dominated by the slower feeding increment, as it is on average more costly and less effective than the slower increment. The cost-effectiveness plane (figure 1) which incorporates the uncertainty around each point estimate in the results shows that relative to the comparator, faster feeding increments are likely to be less cost effective than slower feeding increments (with the scatter plot being predominantly to the left of the origin in figure 1). The CEAC also shows the low probability of the intervention being cost-effective, which clearly decreases as the willingness to pay increases. Thus, in summary, for very preterm or VLBW infants, a faster rate of daily increments in milk volumes (30 mL/kg/day) is probably more costly, on average, at 2 years corrected age and clearly less effective, with fewer infants achieving the principal outcome of survival without moderate to severe neurodevelopmental disability. This result is supported by the data presented in the cost-effectiveness plane for which the majority of the points are in the NW or SW quadrants.

One of the key principles of health economic analysis is to maximise the health benefits from, and ensure the most efficient allocation of, scarce resources. It is plausible to incur analyses that suggest a potentially small decrement in the health outcome is acceptable on cost-effectiveness grounds if the potential cost saving is great enough to more than offset the loss in health outcome and if the saved resources can be used to better effect elsewhere. However, this interpretation does not apply in the current analysis due to uncertainty in a number of areas. First, regarding costs, the PSA and, in particular, the cost-effectiveness plane suggests that faster feeds could be either more or less costly, compared with slower feeds. Second, with respect to the clinical effectiveness, the PSA represented in the cost effectiveness plane (figure 1) indicates a potential risk for harm associated with the faster feeding increment relative to the slower feeding increment based on the distribution of the data at 24 months. All data points are presented in either the NW (more costly, less effective) or SW (less costly, less effective) quadrants. Finally, impaired development at 2 years is a serious outcome associated with life-long consequences and increased costs across a number of sectors including education, healthcare and social care.^2^

There are also broader societal consequences that could result from the clinical outcome, namely productivity loss, stemming from both time off work and the lost earnings of parents of children with disabilities.^1 19^ The trial did not collect resource and outcome data beyond 2 years.

It is noteworthy that while the costs of initial hospital care were lower in those allocated faster increments compared with those allocated slower increments, in the specific period from initial discharge up to 2 years corrected age, the costs incurred by those allocated faster increments were greater overall, per infant, compared with those allocated to slower increments, potentially revealing problems as the infants mature.

In summary, based on neurodevelopmental outcomes at 2 years, and given the uncertainty in both the cost and clinical effectiveness, the faster feeding increment (rate 30 mL/kg/day) that was tested in the trial cannot be advocated on cost-effectiveness grounds.

### Strengths and limitations of the study

Strengths of the economic evaluation include that it is the first, conducted alongside a randomised controlled trial (RCT) comparing enteral feeding practices in infants. SIFT is the largest study of any infant feeding intervention ever undertaken in the neonatal age group and thus there is broad applicability of this economic evaluation, for both policy and practice. The resource use and outcome data were prospectively collected at different points in the trial. Unit costs were obtained from standard and recognised sources. The cost effectiveness results also benefitted from the robustness of the main analyses and stochastic analyses.^20^

A previous clinical systematic review^21^ identified only four RCTs comparing enteral feed volumes in very preterm and VLBW infants. All reviewed studies had a very short follow-up period (2 weeks)^22^ or no follow-up at all.^23 24^ Furthermore, none of these studies included economic evaluations; thus, conclusions could not be made regarding the most cost-effective feeding strategy.

A limitation of this study is that the follow-up period was only 2 years. A longer follow-up would have provided greater scope for observation of the economic consequences of enteral feeding strategies. In particular, the costs and consequences of the disabilities/impairments present and the degree of differentiation in health service use between the two feeding groups would have been informative. Recall bias is another potential limitation as follow-up data were reliant on parent recall over a 24-month period.

Our analyses required some pragmatic assumptions regarding proportions of milk volumes and antibiotic usage during infants’ initial hospital stay due to excessive staff burden in data collection. Nonetheless, our assumptions are supported by existing literature and guidelines, and the relatively low cost impact is unlikely to have any significant impact on the results.

A further potential limitation of this study is the confusion that might arise given that the reported clinical results^20^ showed faster feeds were not statistically significantly different from slower feeds for the primary outcome of survival without moderate or severe disability at 24 months (corrected for prematurity),^20^ whereas the health economics analysis suggests that faster feeding increments cannot be supported on cost-effectiveness grounds as a result of uncertainty in both the costs and outcomes. This contrasting interpretation of the results relates to a requirement in the recommendations for health economic analysis^14^ to estimate and quantify the uncertainty around the clinical endpoints using PSA.^25^ This recommended and widely endorsed approach to conducting robust economic analysis is recognised as potentially challenging and has been widely debated and explained elsewhere.^25 26^

## Conclusion

Based on the results of this within-trial economic evaluation, increasing milk feed volumes at the faster rate (30 mL/kg/day) in very preterm or VLBW infants is not a cost-effective strategy and cannot be recommended.

This work highlights an ongoing debate and also reveals the impact of the difference in paradigms between the statistical approach and economics approach to analysis.^25 27^
