# Supplementary figures and images for: Economic evaluation alongside the Speed of Increasing milk Feeds Trial (SIFT)
Source: Arch Dis Child Fetal Neonatal Ed. 2020 Apr 2;105(6):587–92. doi: 10.1136/archdischild-2019-318346 (PMC7592357; doi:10.1136/archdischild-2019-318346)

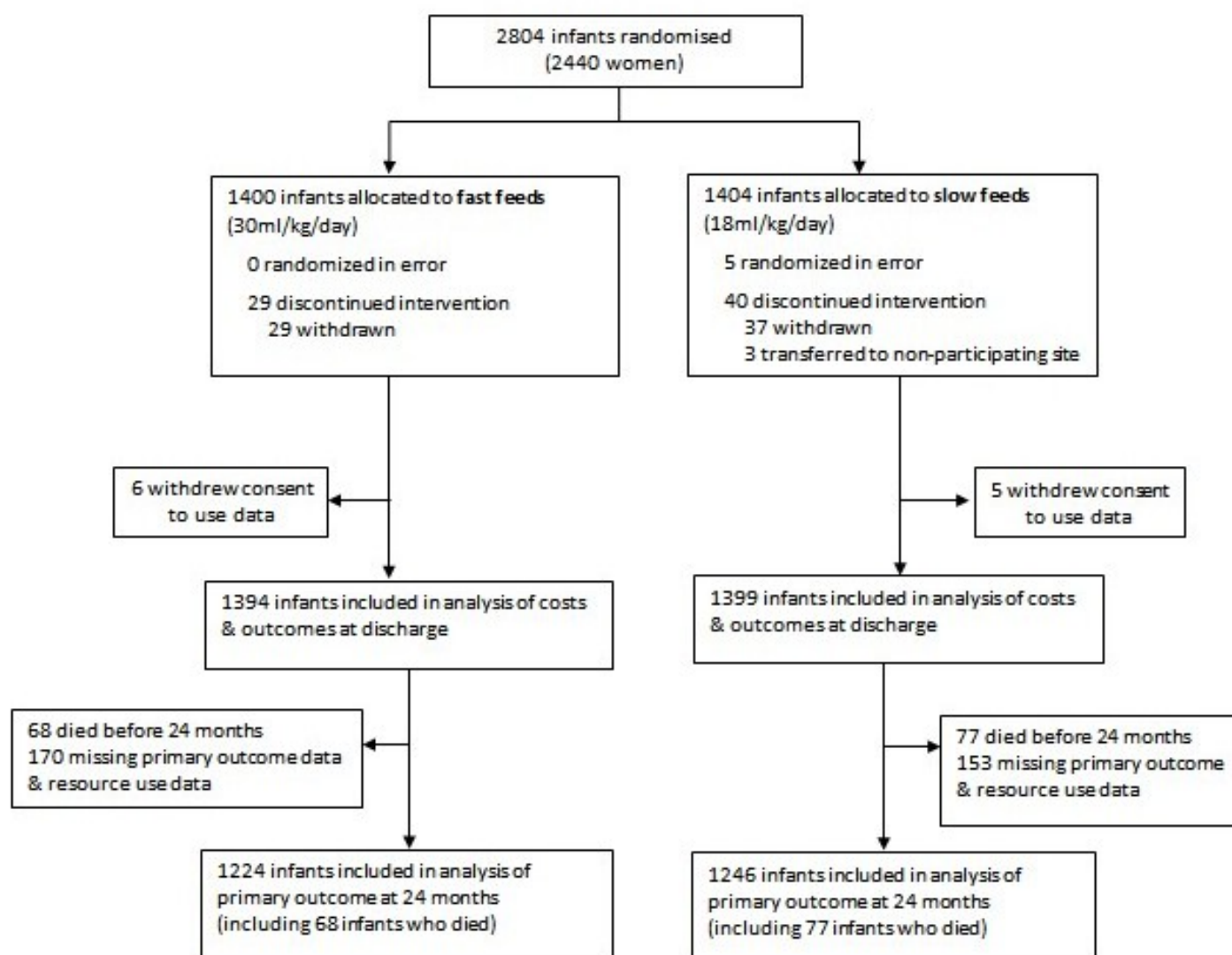

Supplement: Supplementary data [file fetalneonatal-2019-318346supp001.pdf]
