# Supplementary material for: Economic evaluation alongside the Speed of Increasing milk Feeds Trial (SIFT)
Source: Arch Dis Child Fetal Neonatal Ed. 2020 Apr 2;105(6):587–92. doi: 10.1136/archdischild-2019-318346 (PMC7592357; doi:10.1136/archdischild-2019-318346)
Supplement: Supplementary data [file fetalneonatal-2019-318346supp002.pdf]

Supplementary data

Resource items	Faster increments N=1394		Slower increments N=1399		Bootstrap difference (95% CI)	
	Mean	SD	Mean	SD	Adjusted mean difference	95% CI
Days receiving faster or slower feed increments	13.24	16.22	15.04	14.31	-1.80	-2.87 to -0.55*
Days in intensive care						
Level 1 – Intensive Care	15.06	18.43	14.72	17.48	0.34	-0.96 to 1.71
Level 2 – High Dependency Care	20.71	24.79	21.12	19.65	-0.41	-1.96 to 1.51
Level 3 – Special Care	30.13	15.28	29.60	15.05	0.53	-0.58 to 1.68
Initial hospital stay						
Days in hospital	91.00	94.77	94.44	103.76	-3.25	-11.07 to 3.30
Grade 1 IVH/Germinal Matrix Haemorrhage*	0.15	0.38	0.16	0.44	-0.01	-0.04 to 0.02
Grade 2 IVH	0.10	0.36	0.09	0.34	0.006	-0.02 to 0.03
Grade 3 IVH	0.04	0.25	0.03	0.25	0.007	-0.01 to 0.02

Grade 4 IVH	0.04	0.24	0.03	0.25	0.007	-0.02 to 0.02
Shunts for hydrocephalus	0.01	0.13	0.01	0.18	-0.002	-0.02 to 0.009
Bronchopulmonary dysplasia	0.32	0.89	0.31	0.90	0.01	-0.04 to 0.06
Periventricular leukomalacia	0.03	0.27	0.02	0.20	0.009	-0.006 to 0.02
Retinopathy treated medically or surgically	0.07	0.36	0.06	0.32	0.02	-0.002 to 0.04
Patent ductus arteriosus (PDA) treated with NSAID or surgery	0.16	0.49	0.17	0.47	-0.001	-0.03 to 0.03
Surgeries due to gut signs	0.04	0.25	0.04	0.23	0.001	-0.02 to 0.02
Days on antibiotic medication	5.60	11.13	5.55	10.85	0.05	-0.70 to 0.10
Days treated with antifungals	1.20	5.90	1.59	7.52	-0.39	-0.86 to 0.10
Days receiving preterm milk formula	1.74	3.93	1.88	4.14	-0.14	-0.44 to 0.15
Days receiving breast milk fortifier	0.86	2.47	0.83	2.45	0.02	-0.19 to 0.20
Days receiving donated breast milk	1.35	3.34	1.43	3.67	-0.12	-0.41 to 0.11
Days receiving term formula milk	0.24	1.35	0.24	1.57	-0.001	-0.11 to 0.11

* IVH refers to intraventricular haemorrhage (intracranial abnormality)

TABLE S1 Mean resource use across treatment arm

Resource items	Faster increments N=1224		Slower increments N=1246		Bootstrap difference (95% CI)	
	Mean	SD	Mean	SD	Adjusted mean difference	95% CI
Resource use during 2 year follow-up						
Readmissions	0.34	0.48	0.34	0.48	-0.006	-0.04 to 0.03
Operations	1.47	15.71	1.46	0.36	0.02	-0.99 to 1.10
Days as an inpatient	3.23	17.33	2.82	11.84	0.41	-0.60 to 1.73
Routine hospital follow-up visits as a day patient	3.08	6.82	3.17	6.78	-0.1	-0.59 to 0.41
Other hospital outpatient visits as a day patient	0.39	0.49	0.37	0.49	0.02	-0.02 to 0.06
Paediatrician visits as a day patient	1.55	3.96	1.58	3.57	-0.03	-0.29 to 0.26
General Practitioner visits	2.68	6.06	2.42	5.60	0.26	-0.17 to 0.70
Health Visitor appointments	2.05	6.27	1.88	5.86	0.17	-0.26 to 0.61
Community Nurse visits	2.25	21.38	1.28	6.40	0.97	0.13 to 2.61*
Home Visitor/Volunteer visits	0.05	0.21	0.04	0.20	0.004	-0.01 to 0.02

Community Paediatrician visits	0.27	1.46	0.29	1.75	-0.01	-0.14 to 0.11
Physiotherapist visits	2.03	8.00	1.93	8.21	0.1	-0.48 to 0.71
Social Worker visits	0.22	2.02	0.19	2.46	0.03	-0.14 to 0.19
Speech and language therapist visits	0.54	2.70	0.53	2.87	0.006	-0.21 to 0.20
Dietician visits	0.68	3.47	0.73	3.16	-0.05	-0.27 to 0.21

TABLE S2 Disaggregated costs by trial arms (£s sterling, 2017-18 prices)

Resource items	Faster increments N=1394		Slower increments N=1399		Bootstrap difference (95% CI)	
	Mean	SD	Mean	SD	Adjusted mean difference	95% CI
Days receiving faster or slower feed increments	597	731	678	645	-80	-126 to -30*
Days in intensive care						
Level 1 – Intensive Care	19,506	23,863	19,063	22,631	443	-1,272 to 2,277
Level 2 – High Dependency Care	21,378	25,578	21,798	20,280	-420	-2,016 to 1,566
Level 3 – Special Care	15,375	7,793	15,102	7,676	273	-315 to 887
Initial hospital stay						
Grade 1 IVH*/Germinal Matrix Haemorrhage	127	331	134	381	-11	-38 to 15
Grade 2 IVH	143	532	136	494	7	-28 to 46
Grade 3 IVH	60	378	50	381	10	-19 to 40
Grade 4 IVH	54	364	51	366	4	-23 to 30
Shunts for hydrocephalus	24	348	28	477	-4	-40 to 24
Bronchopulmonary dysplasia	2,475	3,970	2,392	4,112	83	-214 to 362

Resource items	Faster increments N=1394		Slower increments N=1399		Bootstrap difference (95% CI)	
	Mean	SD	Mean	SD	Adjusted mean difference	95% CI
Periventricular leukomalacia	48	305	36	240	12	-7 to 32
Retinopathy treated medically or surgically	137	499	103	453	35	-1 to 70
Patent ductus arteriosus (PDA) treated with NSAID or surgery	202	509	202	537	0.58	-37 to 43
Surgeries due to gut signs	237	1567	231	1,536	5	-116 to 114
Days on antibiotic medication	16	33	16	32	0.16	-2 to 3
Antifungals	1	6	2	8	-0.41	-0.89 to 0.14
Preterm milk formula	0.04	0.08	0.04	0.09	-0.003	-0.009 to 0.004
Breast milk fortifier	0.79	2	0.78	2	0.02	-0.17 to 0.20
Donated breast milk	438	1,120	480	1,232	-41	-127 to 51
Term formula milk	0.37	2	0.37	2	-0.001	-0.19 to 0.15
Resource use during 2 year follow-up	**N=1224**		**N=1246**			
Operations	3,316	35,294	3,273	29,567	42	-2,259 to 2,612
Inpatient stays	2,150	11,057	1,883	7,577	267	-326 to 1,076

Resource items	Faster increments N=1394		Slower increments N=1399		Bootstrap difference (95% CI)	
	Mean	SD	Mean	SD	Adjusted mean difference	95% CI
Outpatient visits	1,067	1,971	1,082	1,827	-18	-162 to 112
General Practitioner visits	88	200	80	185	9	-6 to 24
Health Visitor appointments	154	471	141	440	13	-23 to 45
Community Nurse visits	81	770	46	230	35	5 to 94*
Home Visitor/Volunteer visits	0.86	4	0.77	4	0.09	-0.20 to 0.40
Community Paediatrician visits	112	596	117	712	-6	-59 to 39
Physiotherapist visits	193	760	183	780	9	-55 to 61
Social Worker visits	9	79	8	96	1	-6 to 7
Speech and language therapist visits	51	257	50	273	0.59	-19 to 18
Dietician visits	58	295	62	269	-4	-23 to 19

* IVH refers to intraventricular haemorrhage (intracranial abnormality)

TABLE S3 Mean costs calculated with multiple imputation (£s sterling, 2017-18 prices)

Cost	Faster feeds (n=1394)		Slower feeds (n=1399)	
	Mean	SD	Mean	SD
**Total costs of health service use after initial discharge from hospital and up to 24 months corrected age**	109,410	92,266	109,032	89,763
